# Opinions of Prospective Dentists and Prospective Teachers on Antibiotic Use

**DOI:** 10.3390/healthcare10122527

**Published:** 2022-12-14

**Authors:** Seyda Gul, Pinar Gul

**Affiliations:** 1 Kazım Karabekir Faculty of Education, Ataturk University, Erzurum 25240, Turkey; 2 Department of Restorative Dentistry, Faculty of Dentistry, Atatürk University, Erzurum 25240, Turkey

**Keywords:** antibiotics, university student, unreasonable consumption

## Abstract

The purpose of this study was to examine the opinions of prospective dentists and prospective teachers on antibiotic use. In this study, a survey method based on a quantitative research paradigm was utilized. A 19-item instrument was applied to the sample group including 414 university students attending to the Faculty of Dentistry and Faculty of Education. The instrument was composed of three main subscales in addition to demographic characteristics. The first part focused on attitudes, the second part focused on subjective norms, and the third part focused on the prospective dentists and prospective teachers’ intentions regarding the phenomenon of antibiotics use. While the dependent variables of the study were the participants’ levels of agreement in the subscales of the instrument, the independent variable was faculty studied. The data were analyzed through descriptive statistics, and the Mann–Whitney U test was used to compare the participants’ views on antibiotic use. The findings in terms of the faculties showed that dentistry and education faculties in our country prepare well in expanding their reasonable consumption of antibiotics. Yet, antibiotics are used more incorrectly by some prospective teachers than by prospective dentists. According to the findings in terms of subscales, the participants have positive attitudes toward the reasonable use of antibiotics in terms of the attitude subscale. However, the results from the subjective norm indicated that participants are influenced by their environment in their antibiotic use. Finally, the results from the intention indicated that participants avoid using antibiotics irregularly or frequently.

## 1. Introduction

Antibiotics are among the indispensable drugs of modern medicine, as these drugs stop infections by killing the bacteria that cause the infection or preventing them from spreading in the body. Thus, they help the host defend itself and eliminate bacteria [1,2,3,4]. In this way, they have significantly reduced deaths from infectious diseases and improved survival [2].

Antibiotics are used in human and animal health as the treatment of infectious diseases, in the preservation of food in the food industry, and in scientific research activities in hospitals and the pharmaceutical industry [5]. However, antibiotics are the most widely consumed and misused drug group. Unreasonable antibiotic consumption is common, especially in developing countries [6]. Unreasonable antibiotic consumption can be defined as the use of an excessive number of drugs for patients, the use of inappropriate antibiotics, the prescribing of drugs that do not comply with clinical guidelines, non-compliance with the recommended drug treatment by patients, and the patients’ efforts to treat themselves inappropriately [7,8]. Unreasonable consumption of antibiotics can increase the risk of antibiotic resistance and reduce the effectiveness of antibiotics used in the treatment of infections caused by bacteria [6]. Hence, it is an important public health problem that causes the rapid spread of resistant bacteria in society [5,9,10,11,12].

Antibiotic resistance, defined as the ability of bacteria to resist the effects of antibiotics, is one of the biggest factors threatening human health [1]. Unreasonable or excessive consumption of these drugs, self-medication, and incorrect prescription can be important factors in both the increase and spread of antimicrobial resistance [13,14]. Especially self-medication causes drug reactions, antibiotic resistance, and waste of public expenditures [5,15]. 

According to the data of from the World Health Organization (WHO), Turkey is the country using the most antibiotics in Europe based on the frequency of use of antibiotics [7,16]. This may be an indication that antibiotics, which have an important place in drug consumption in our country, are used unconsciously. In recent years, efforts to control the use of antibiotics have started to gain momentum in order to minimize the negative consequences of unnecessary and unreasonable use of antibiotics in the world and our country [17,18]. Many strategies have been proposed around the world, such as developing or restricting formulas for antibiotic use, training healthcare professionals, obtaining the approval of a specialist in taking drug prescriptions, and the reasonable use of antibiotics [19,20,21]. However, it is likely that the knowledge, attitude, and awareness of individuals toward antibiotics use will be affected, especially with the COVID-19 outbreak. Therefore, students, teachers, and researchers in general, as well as healthcare personnel, should greatly support education in the judicious use of antibiotics. Undoubtedly, education is one of the effective ways to increase the awareness of individuals about the judicious use of antibiotics [22,23]. Universities are one of the most basic institutions where training on antibiotic use can be given. As a matter of fact, the WHO’s Global Action Plan on Antimicrobial Resistance is recommended to organize training programs to develop awareness of society to use the correct medication, in line with this view [17,21]. Additionally, the WHO has recently stressed the importance of undergraduate education in prudent prescribing [24]. Therefore, it is important to provide training to both the public and health personnel on the judicious use and prescribing of such drugs [25,26]. On the other hand, in studies conducted at different levels of education, students’ lack of knowledge and irrational use of antibiotics have been identified. Therefore, it is crucial that students be educated about the judicious use of antibiotics as the antibiotic users of tomorrow [26,27]. However, although the health personnel receive training for this awareness in our country, it is a major deficiency especially in the faculties of education that train teachers [27]. Except for biology or science teaching departments, this subject is ignored in the curricula of other branches. In other words, science educators and practitioners are partially involved in the introduction of antibiotics in schools, and this interest is more or less reflected in the curriculum of teacher training programs, especially science and biology education. As a result, curricula can be updated for some specific courses of such everyday life-related concepts, not only in science but also in other fields. Thus, effectively teaching the judicious consumption of such drugs can help students understand the risks, benefits, and disadvantages of antibiotic use as well as increase their knowledge. For this reason, creating positive behavioral changes related to the use of antibiotics in individuals has an important place in minimizing negative consequences. One way to achieve this is to examine individuals’ knowledge, attitudes, or views on antibiotic use [1,9,23,24,28,29].

There are many studies in the literature investigating the knowledge of university students on rational antibiotic use and antibiotic resistance in different countries [27,29,30]. Similarly, many studies were also conducted so far among nursing and medical students and patients/local people in Turkey [11,16,17,22]. However, there are only a few studies conducted with dental students [31]. Given that the cooperation between educational and health partners is clear [26], understanding the knowledge, attitudes and practices of both dental students and prospective teachers about antibiotic use can greatly affect the problems related to antibiotic use. On the other hand, fewer concerns are focused on dental students and prospective teachers [32]. The insufficiency of studies examining the opinions of prospective dentists and prospective teachers on antibiotic use in our country highlights the importance of conducting this study for adequate decision making. Hence, this study aims to examine the opinions of prospective dentists and prospective teachers on antibiotic use.

The null hypothesis of the study is “There is no difference between the opinions of dentists and teacher candidates about antibiotic use”.

## 2. Materials and Methods

In this study, a survey method based on a quantitative research paradigm was utilized. In survey research, the researcher applies a survey to a particular sample or conducts interviews to gather information about the subject he/she is researching. Surveys are used to learn different information about people’s attitudes, beliefs, values, demographics, behaviors, opinions, habits, etc. [33].

### 2.1. Ethics Procedure

All procedures performed in the study were in accordance with the ethical stand-ards of Atatürk University. Ethics approvals were obtained with the decision (reference no: E-56785782-050.02.04-2200126787) of the Social and Human Sciences Ethics Committee. Likewise, the participants in this study completed and signed an informed consent form detailing the entire procedure and the protection of their data. 

### 2.2. Sample

The sample group included 414 university students attending to the Faculty of Dentistry (119 female, 81 male) and the Faculty of Education (161 female, 53 male) at a university in Turkey (Table 1). In determining the sample, the rule of thumb according to Bryman and Cramer is that [34] “the sample should be at least five times the item number in the scale”. Additionally, G*Power 3.1.9.4 software (Heinrich-Heine Dusseldorf University, Dusseldorf, Germany) was used to determine the sample size based on using the following parameters: 95% power, 0.36 effect size, and α error at 0.05. A minimum sample size of 404 participants was assessed to be appropriate.

The data could not be collected through traditional paper-and-pencil methods because of the COVID-19 outbreak, which created educational disruptions [35]. In this process, the researchers took some measures to reduce the social desirability bias in the survey. For this, first of all, an online AUS was created with Google Forms (no in-person contact). In addition, descriptive information other than the gender, class level and faculty of the participants was not included in the AUS. Alongside ensuring anonymity, each participant was informed that their responses will never be disclosed to any third party. Students were asked to voluntarily answer the questionnaire. On the days of data collection, all students from the faculty of dentistry and education were invited to participate in the study. All researchers of the study contacted the students and explained the purpose of the study. No incentive was offered to complete the AUS, and each student was free to refuse to participate in the study. Data were collected anonymously through an online questionnaire. The questionnaire has been prepared in such a way that it cannot be completed without marking all the questions. For this reason, there were no missing or empty answers in any of the collected data. Since the questionnaires were filled voluntarily, 1st and 2nd year students participated more in both faculties. Due to the imbalance in the number of students according to both gender and class, the hypothesis of the research was designed only according to the faculty variable.

### 2.3. Data Collection Tool and Analysis Process

The data were collected through a personal information form and Antibiotic Use Scale (AUS) developed by Atik and Doğan [5]. The personal information form was prepared by the researchers to identify the demographic characteristics of participants, such as gender and faculty. Permission was obtained from the researchers who developed the AUS for the applications. During the development process of AUS, a trial form was prepared by Atik and Doğan [5], and expert opinions were received. Thus, there were 53 items in the trial form. For construct validity, the trial form was applied to 424 university students, and explanatory factor analysis (EFA) was performed. In addition, confirmatory factor analysis (CFA) was conducted on 212 students. After factor analysis, 3 factors (subscales) with eigenvalues greater than 1 and explaining 58.565 of the total variances were determined. AUS included 19 items with three subscales, namely Attitude (11 questions), Subjective norm (5 questions) and Intention (3 questions). On the other hand, the item total score correlations and Cronbach Alpha internal consistency coefficient were examined for the reliability of the scale. It was revealed that the item–total score correlations of the items in the scale varied between 0.601 and 0.785. These findings showed that the items differentiated individuals well. Reliability coefficients of the AUS were also calculated. While the Cronbach Alpha internal consistency coefficient was found to be 0.945 for the overall scale, it was calculated as 0.929 for the attitude subscale, 0.836 for the subjective norm subscale and 0.854 for the intention subscale. In addition, the Spearman Brown correlation coefficient of the scale was calculated as 0.925. In this study, the Cronbach Alpha internal consistency coefficient was found to be 0.928 for the overall scale, 0.926 for the attitude subscale, 0.761 for the subjective norm subscale, and 0.865 for the intention subscale. According to these reliability coefficients obtained for the developed scale, it is considered as a scale with high reliability.

AUS included 19-items with three subscales namely Attitude (11 questions), Subjective norm (5 questions) and Intention (3 questions). AUS was prepared in a five-point Likert type (1 = Strongly disagree, 2 = Disagree, 3 = Neutral, 4 = Agree, 5 = Strongly agree), and the students were asked to state their agreement level for each item in AUS. The data was recorded in the Google Form and then transferred to Microsoft Excel. Then, the data obtained in the study were analyzed in the SPSS 20.0 statistics program and *p*-values of <0.05 were considered significant. In the analysis, descriptive and inferential statistics were conducted. The means were interpreted as follows: strongly disagree in the point range of 1.00–1.80, disagree 1.81–2.60, neutral 2.61–3.40, agree 3.41–4.20, and strongly agree 4.21–5.00.

Since the participants answered the questionnaire voluntarily, the uneven distribution of the participants’ class level and gender was remarkable. For example, as the majority of the survey population were female participants, survey scores were not compared according to the gender variable. On the other hand, education is given for 5 years in the faculty of dentistry and for 4 years in the faculties of education. For this reason, besides the difference in the grade levels of both faculties, there was no equivalence in the number of students in each class. Since this situation may negatively affect the research results, only the faculty variable was considered in the comparisons, and therefore, the research hypothesis was prepared only according to the faculty variable. Non-parametric statistical techniques were used for comparisons made in terms of the faculty. The Mann–Whitney U test was used for pairwise comparisons, and the significance value was taken as *p* < 0.05 in the analysis of the data.

## 3. Results

In this study, the descriptive statistics regarding the participants’ opinions toward antibiotic use are presented in Table 2.

As shown in Table 2, it is observed that the ones with the best scores correspond to the subscale “Intention”. However, when the means were examined, the participants stated that they did not agree with the items both in general and in subscales. Considering the structure of the related items, the “disagree” opinions of the participants can be evaluated positively.

As a result of the analyses performed to answer the null hypothesis of the research, the participants’ opinions on antibiotic use were compared in terms of faculties. For this aim, in order to decide which statistical techniques will be used to answer the research hypothesis, the distribution of the mean scores of the AUS was examined. Using the Kolmogorov–Smirnov and Shapiro–Wilk tests, the conformity of the data to the normal distribution was checked, and the results of the analysis revealed that the data structure did not meet the assumptions of the parametric test (Table 3).

According to the findings shown in Table 3, the Mann–Whitney U test was performed to determine whether the opinions of the prospective dentists and teachers differ in terms of faculties. The results of the analyses were presented in Table 4.

According to the findings in Table 4, it is seen that there is a significant difference between the scores in the subscale ‘Attitude’ (U = 17748.000, *p* < 0.05) and the scores of the overall scale (U = 18581.000, *p* < 0.05) in terms of faculties. In other words, the prospective teachers’ agreement levels with the statements on the scale are higher than the prospective dentists. Additionally, effect size values were calculated as η^2^ = 0.022 for the attitude subscale, η^2^ = 0.005 for the subjective norm subscale, η^2^ = 0.000 for the intention subscale, and η^2^ = 0.012 for the overall scale. Cohen suggested that small, medium, and large effects would be reflected in values off equal to 0.10, 0.25, and 0.50, respectively [36]. According to this, it can be said that the effect size of faculty is quite small.

## 4. Discussion

According to the finding from this study, dentistry and education faculty students were aware of reasonable antibiotic consumption. For this reason, it can be said that dentistry and education faculties in our country are well prepared to promote reasonable antibiotic consumption. This finding shows that contrary to many studies in the literature [2,21,24], the awareness level of university students regarding antibiotic use has increased. However, according to the results of the analysis, it was determined that there was a statistically significant difference between faculties in terms of general averages of AUS. Accordingly, it can be said that prospective dentists are more conscious about rational antibiotic use. In fact, this finding may be related to the professions of dental students. On the other hand, when the effect size values are examined, it has been determined that the effect of the faculty is quite small both in the whole scale and in all subscales. Therefore, both the effect size courses and the average values reveal that this awareness should be increased a little more for all students. Of course, it would be useful to examine the findings in terms of students’ grade levels in order to reach a more precise judgment on this issue. Because the higher the level of education provided, the more likely the knowledge of students will increase. Indeed, studies in the literature have found that knowledge of antibiotics and antibiotic resistance increases with successive years of faculty education [37]. In this study, since most of the participants were students who were just starting their education, no comparison was made according to years, considering that this could affect the findings of the study.

When the findings are examined in terms of subscales, similar results were found. In the attitude subscale, the opinions of the participants about the results of antibiotic use and the statements in which the possible consequences are evaluated by the individual are included. The findings indicated that prospective dentists were more conscious of the use of antibiotics than prospective teachers. This situation may be related to the competencies of prospective dentists in the field of health education. As a matter of fact, in the study conducted by Atik and Doğan [5], it was determined that teacher candidates’ attitudes toward rational antibiotic use were low. In this regard, it is important to remember that the WHO has highlighted the importance of improving the training of undergraduate students with antibiotic use as one of the main strategies to preserve the effectiveness of antibiotics [2]. On the other hand, although there is a statistically significant difference between the faculties in the attitude subscale, the AUS showed that a significant proportion of respondents in both faculties disagreed with antibiotic use. This finding can be interpreted as an indication that the participants have positive attitudes toward the reasonable use of antibiotics. Students do not prefer to use antibiotics frequently, especially in viral infections such as flu or in different diseases. At the same time, participants do not believe that using frequently antibiotics is beneficial and do not recommend it to the people around them. On the contrary, the findings from Aljayyousi et al. [1] indicated that a significant proportion of participants had a negative attitude toward antibiotic use. According to the findings, the majority of the participants claimed that antibiotics are effective against viral infections, and antibiotics speed up the recovery from most coughs and colds. Findings regarding the use of antibiotics against coughs and colds have been demonstrated in many studies [6,22,27,38,39]. As a result, contrary to the findings in the literature, it is seen that the participants in this study have positive attitudes toward the correct use of antibiotics, and their awareness of the use of antibiotics is high. This situation, in parallel with the coronavirus disease (COVID-19) pandemic, may cause individuals to increase their awareness level, especially about flu infections, and therefore be more cautious about the use of antibiotics. Similarly, the study of Nymand et al. [39] found that COVID-19 restrictions in Denmark contributed to both lower antibiotic consumption and a change in prescribing patterns in general practice. Thus, it is likely that some of the initiatives to prevent COVID-19 may be important prospectively in the judicious use of antibiotics and in the fight against antimicrobial resistance.

According to the findings from the subjective norm subscale, it was detected that there was no statistically significant difference found in terms of faculties. According to the findings, a significant proportion of participants disagreed with the statements in AUS. When the means in the subjective norm subscale were examined, it seemed the students did not agree with the items. For example, the majority of the participants do not buy the antibiotic from any pharmacy without a prescription or without consulting a doctor. One of the possible reasons for this situation may be related to the health system in our country. In 2003, radical changes were made in the health system with the Health Transformation Program. Parallel to the implementation of the program, the General Health Insurance system, which covers everyone, was introduced. In order to increase accessibility to health services, preventive health services have been started to be provided free of charge. Changes were made for the efficient use of resources, eliminating inequalities in access to health services and effective use of health services [40]. Even low-income citizens can benefit from health services with government support. In addition, the increase in the number of hospitals, the improvement of the health system, and the easy access to doctors may have contributed to the increase in antibiotic awareness. Similarly, Bayram et al. indicated that the attitudes of the participants to consult a doctor, follow recommendations and read instructions were found in over 85% [10]. Dyar et al. found that most students agreed on which antibiotic prescribing was inappropriate and considered it professionally unethical [21]. According to Dyar et al., the link between improper prescribing and ethical responsibility may be another way of involving students in management efforts, as most students believe that prescribing will help somewhat avoid the problem of antibiotic resistance [21].

On the other hand, in the subjective norm subscale, there are statements about the effect of the opinions of people who are considered important by the participants on their use of antibiotics. A high total score on this dimension will mean that they are influenced by their environment in their antibiotic use. According to other expressions in the subjective norm subscale, it can be said that the participants are somewhat affected by the people around them regarding the use of antibiotics. Similarly, in the studies implemented by Aljayyousi et al., Çelik et al., and Özkan et al., the majority of the participants claimed which it is not wrong to use antibiotics with the advice of friends or relatives without proper medical advice [1,17,41]. In conclusion, these findings show that even if patients receive the treatments recommended by physicians, it is their decision-making mechanisms that determine drug use. These decisions are influenced by the knowledge gained, the beliefs of family, friends or society, and encouraging suggestions [37,42,43,44].

According to the findings from the intention subscale, it was detected that no statistically significant difference was found in terms of faculties. At the same time, it was founded that a significant proportion of participants disagreed with the statements in AUS. The intention dimension is the most important predictor of the antibiotic use behaviors of the participants. In other words, it can be said that the higher the average of the total intention items, the more determined the individual is to use antibiotics. In this case, the fact that the participants did not agree with the statements indicates that they avoid using antibiotics irregularly or frequently. However, although the students did not agree with the statements, the rate of agreement with the statements in the intent subscale is slightly higher when compared to the other subscales of the AUS. In this case, ease of access to drugs may be one of the possible reasons for this situation. The findings partially parallel the studies in the literature [27,37,40,45]. For example, Hu et al.’s study showed that most students stockpile high levels of antibiotics and self-medicate. They also found that a quarter of them used antibiotics unnecessarily for a self-limited disease [6].

This study has several limitations: (i) The questionnaire was applied to students via e-mail during the pandemic period and was voluntarily. Therefore, participation in the survey was low. (ii) We are unsure of the diversity of structure, content and learning pedagogy that could influence responses across all the two faculties included. However, the responses were consistent for most questions in both the dental and education faculties studies, indicating some degree of external validity. (iii) On the other hand, although one of the reasons for the increase in antibiotic awareness according to the findings of the study is that the questionnaire was applied during the pandemic process, the possible impact of the pandemic could be understood more clearly if the application was carried out before the pandemic and the findings were compared. (iv) Another limitation of the study is that the survey scores were compared only in terms of faculties, since the number of students in terms of gender and grade levels is not close.

## 5. Conclusions

Within the limitations of this study, although students’ awareness of appropriate antibiotic use has increased compared to previous studies in our country, unreasonable antibiotic use continues, especially among teacher candidates. In this case, awareness can be increased by giving lessons to each student before they come to the university. Projects can be carried out to further increase cooperation between education faculties of universities and faculties of health sciences, including dentistry. In addition, training seminars can be given not only to university students but also to lower levels of education. There is a need to raise awareness about the rational use of antibiotics in society and to inform society continuously. For this, efforts should be made to involve especially teachers more in the process as well as to make more use of and development of formal and non-formal education opportunities. For example, it may be beneficial to include applied activities on the use of antibiotics in curricula, in science courses.

## Figures and Tables

**Table 1:** Demographic characteristics of the sample group.

Characteristics		N (%)
Faculty	Faculty of Education	214 (51.7%)
	Faculty of Dentistry	200 (48.3%)
Gender	Female	280 (67.6%)
	Male	134 (32.4%)
Grade level	1st grade	110 (26.6%)
	2nd grade	144 (34.8%)
	3rd grade	38 (9.2%)
	4th grade	68 (16.4%)
	5th grade	54 (13.0%) *

* Education faculties provide education for 4 years.

**Table 2:** AUS scores related to the participants’ opinions towards antibiotic use.

Factor	Item	Mean	SD	Agreement Level
Attitude	Whenever I’m sick, I can’t get better without antibiotics.	1.80	0.89	strongly disagree
	I recommend those who have flu and cold use antibiotics.	1.80	0.87	strongly disagree
	I believe that taking antibiotics every time I get sick will be beneficial.	1.54	0.78	strongly disagree
	I feel more comfortable when I take antibiotics.	2.12	1.04	disagree
	Being sick makes me very unhappy, and I want to use antibiotics to get better as soon as possible.	1.88	0.94	disagree
	Like some people in the community, I believe that taking antibiotics is always helpful.	1.53	0.78	strongly disagree
	The thought that I will recover faster when I use antibiotics relieves me.	2.12	1.04	disagree
	Like the people around me (my family/friends, etc.), I believe that it is not possible to get well without taking antibiotics in cases such as colds, and flu.	1.61	0.85	strongly disagree
	I always think that the use of antibiotics in cases such as a cold will speed up the recovery.	2.00	0.97	disagree
	I believe I will get better sooner when I use antibiotics.	2.30	1.04	disagree
	I believe I will suffer less when I use antibiotics.	2.11	0.99	disagree
**Total**		**1.89**	**0.71**	**disagree**
Subjective norm	If my friends give antibiotics for any cold and flu, I will accept their offer.	1.50	0.83	strongly disagree
	I don’t mind using the antibiotic given by the pharmacist.	2.44	1.15	disagree
	I do not see any harm in using antibiotics on recommendation without consulting a doctor.	1.48	0.85	strongly disagree
	My parents think it’s okay for me to take antibiotics to get better with colds and flu.	2.38	1.11	disagree
	If my acquaintances, such as a trusted family member or friend, recommend it, I don’t mind using antibiotics.	1.74	0.89	strongly disagree
**Total**		**1.91**	**0.70**	**disagree**
Intention	I save the remaining antibiotics for future reuse.	2.03	1.17	disagree
	After use, I keep any antibiotics left at home for reuse.	2.29	1.20	disagree
	When I have similar complaints, I do not hesitate to use the antibiotic I used before without a prescription.	1.94	1.06	disagree
**Total**		**2.09**	**1.02**	**disagree**
**Overall**		**1.93**	**0.65**	**disagree**

**Table 3:** Normality test results in terms of gender and faculty.

Variable		Kolmogorov–Smirnov ^a^			Shapiro–Wilk		
		Statistic	df	*p*	Statistic	df	*p*
Faculty	Faculty of Education	0.067	214	0.022	0.967	214	0.000
	Faculty of Dentistry	0.085	200	0.001	0.946	200	0.000

^a^. Lilliefors Significance Correction.

**Table 4:** Mann–Whitney U test results for the AUS scores according to faculties.

Groups	Test Statistics	Attitude	Subjective Norm	Intention	Total
Faculty of Education	Mean rank	224.57	214.62	203.38	220.67
Faculty of Dentistry		189.24	199.88	211.91	193.41
	U	17,748.000	19,876.000	20,517.500	18,581.000
	Z	−3.009	−1.259	−0.738	−2.319
	***p***	0.003 *	**0.208**	**0.460**	0.020 *

* *p* < 0.05.

## Data Availability

Not applicable.
